# Thermal imaging demonstrates physiological responses to grooming interactions and audience effects in wild baboons

**DOI:** 10.1098/rsos.251398

**Published:** 2025-10-15

**Authors:** Eleane Jao, Elise Huchard, Elisa Fernández Fueyo, Julia A. Kunz, Guy Cowlishaw, Alecia Carter

**Affiliations:** ^1^ Department of Anthropology, University College London, London, UK; ^2^ Biologie Evolutive Humaine, Institut des sciences de l'evolution, Montpellier, Languedoc-Roussillon, France; ^3^ Gobabeb Research and Training Centre, Walvis Bay, Namibia; ^4^ Department of Biological Sciences, Royal Holloway University of London, Egham, UK; ^5^ Universite de Montpellier, Institut des Sciences de l'Evolution de Montpellier, Montpellier, Occitanie, France; ^6^ Department of Evolutionary Anthropology, University of Zurich, Zurich, Switzerland; ^7^ Institute of Zoology, London, UK

**Keywords:** *Papio ursinus*, thermography, physiology, grooming, emotion

## Abstract

Infrared thermography is an emerging non-invasive tool for monitoring physiological responses to stimuli, yet its application in wild settings has been limited. This study quantified variation in nasal temperatures via thermal imaging of wild chacma baboons (*Papio ursinus*) in Namibia. We aimed to determine whether social and solitary activities exhibit distinct physiological responses and explore the potential of thermal imaging in wildlife behavioural studies. We collected 1626 thermal images of 105 individuals during the baboons’ natural behaviours. Using linear mixed-effects models, we show that, compared with foraging and resting, nasal temperatures were elevated in baboons during grooming, which may indicate relaxation. Additionally, we find that the presence of more neighbours and higher ranking neighbours is associated with cooler nasal temperatures, suggesting an audience effect. These findings further demonstrate that infrared thermography can quantitatively link differences in observed behaviours to associated physiological states through fine thermal cues in unrestrained primates. Thermal biomarkers have the potential to inform social processes, welfare states, emotions and stress responses in wildlife behaviour studies.

## 1. Introduction

Recent thermal imaging technology has opened up the possibility of studying aspects of animal emotion through physiological responses, measured through facial temperature changes [1–5]. Unlike emotions, feelings are the conscious, subjective experiences of emotional states, accessible in humans through verbal reports [6]. While it is impossible for wild animals to answer questions about how they feel, researchers can instead focus on studying emotions, which can be examined through observable physiological and behavioural manifestations [7]. Thermal imaging allows us to observe fluctuations in nasal temperature, which can serve as an indicator of physiological arousal, stress [8]. For example, relaxation, a common socio-positive experience [9], is linked to subcutaneous vasodilation mediated by parasympathetic activation that can be measured with an increase in nasal temperature [10]. By contrast, negative experiences that may result in psychological distress activate a sympathetic response involving vasoconstriction that can be quantified through decreased nasal temperatures in primates [3,11]. Laboratory studies have confirmed the physiological response to negative emotional stimuli: when given threatening visual [5] or auditory stimuli [4], nasal temperature decreased in captive rhesus macaques. Further evidence linking nasal temperature to experience comes from observations of changes in facial temperature during ‘naturalistic’ experimental settings. For example, the nasal temperature of captive rhesus macaques while playing was higher than when feeding [3]. This suggests that rhesus macaques perceive playing as a positive experience and foraging near conspecifics or being teased as negative experiences, perhaps because these are perceived as competitive behaviours. In the context of feeding, competition for resources can introduce stress and rivalry among individuals, leading to lower nasal temperatures as a physiological response to the competitive pressure [1].

While emotions are typically defined as short-term affective states that involve coordinated physiological and behavioural changes [12], our focus here is not on identifying specific emotions *per se* but rather on quantifying observable physiological responses, such as facial temperature changes, as manifestations of arousal, stress, or relaxation. These responses offer insight into the animal’s internal state without relying on assumptions about their subjective experience. A range of physiological measures, including hormone levels, heart rate and peripheral blood flow can provide insights into underlying emotional responses. In both captive and wild primates, affiliative behaviours like grooming have been linked to reduced physiological markers of stress, such as decreased heart rate, cortisol levels and lower baseline glucocorticoid levels [13,14]. By contrast, competitive or tense contexts, such as foraging near dominant individuals or after aggression, are associated with increased glucocorticoids and elevated heart rate, reflecting higher arousal and negative emotional valence [15,16]. These physiological changes are driven by autonomic processes, which are the involuntary regulation of bodily functions, such as heart rate and blood flow, that also influence surface facial temperature, supporting the rationale for using thermal imaging to assess emotional responses.

Building on this physiological foundation, thermal imaging is increasingly being used to investigate how animals respond to social contexts in wild settings, offering a novel, non-invasive window into their physiological and, possibly by extension, emotional states. For instance, wild chimpanzees had lower nasal temperatures when foraging with more competitors, i.e. adult males, nearby [1], which may indicate an activation of their sympathetic nervous system preparing them to respond to potential threatening events. Similarly, chimpanzee males who compete for access to receptive females had lower nasal temperatures when copulating, and particularly when copulating furtively, indicating heightened arousal or stress. By contrast, during cooperative activities that strengthen social bonds (i.e. playing, patrolling and grooming), nasal temperatures were higher, consistent with a relaxed, positive emotional state [2]. These findings align with known autonomic responses and provide growing support for the use of nasal temperature as a physiological proxy for emotion in non-human animals. While cross-species comparisons must be interpreted with caution, these converging observations offer a promising foundation for investigating emotional processes in wild animals through thermal imaging. However, this approach still requires validation across a broader range of species and behavioural contexts.

Apart from studies that gave insight into how chimpanzees perceive their social environment, few thermal imaging studies have been conducted in natural settings and few other species have been considered [1,2,17–19]. Our aim was to expand this field by studying wild chacma baboons’ (*Papio ursinus*) nasal temperatures in different social and behavioural contexts. The chacma baboon is an excellent model species to investigate emotional responses to social stimuli using thermal imaging: it lives in large, stable social groups whose members interact with each other daily; it habituates readily to observers on foot, allowing relatively close approaches for thermal photography and it is largely terrestrial allowing excellent visibility. Chacma baboons have strict and linear dominance hierarchies, where females’ ranks influence their access to resources and reproductive success [20]. Female chacma baboons inherit their social rank and relationships from their mothers, and social interactions are generally clustered within matrilines [21]. They may use grooming to ‘trade’ for access to food or infants and to strengthen social bonds [22]. Male chacma baboons fiercely compete through fighting to gain higher dominance rank and to access females for mating [23].

We had four specific aims under our overarching objective to determine whether nasal thermal imaging could serve as an indicator of real-time physiological responses to activities and social conditions in wild chacma baboons. The four aims were to: (i) determine whether field measurements of nasal temperatures under natural conditions are reliable; (ii) investigate whether baboons show a physiological response, measured through differences in nasal temperature, during different activities; (iii) determine whether giving or receiving grooming has a different effect on nasal temperature, that could be indicative of a differential emotional salience; and (iv) assess whether there is an audience effect on baboons’ nasal temperatures during foraging, resting and grooming. We predicted that (1) thermal images would be repeatable within a ‘session’, i.e. images taken of the same baboon in the same activity state within a short period of time would show the same nasal temperatures with marginal variations [24]. We further predicted that (2a) foraging may induce stress because of competition, resulting in lower nasal temperatures recorded during foraging compared with resting and grooming [1,2,4,5,25], and (2b) grooming, a socio-positive interaction [9,10], would elicit a relaxed state characterized by higher nasal temperatures compared with resting and foraging. We predicted that (3) individuals receiving grooming (groomees) would be more relaxed and have a more pronounced increase in nasal temperature compared with those giving grooming (groomers) [2]. We further predicted that (4a) a larger audience and (4b) an audience including higher ranking individuals would be more stressful and result in a lower nasal temperature of the focal individual than smaller audiences composed of lower ranking individuals [1,2]. Finally, we predicted that (4c) individuals with stronger social bonds to audience members would have an increased nasal temperature, indicating a more relaxed state when they range around familiar individuals [26].

## 2. Methods

### 2.1. Study site and population

The study was conducted at the Tsaobis Nature Park and surrounding farmland, Erongo region, in central Namibia (15°45′ E, 22°23′ S). Tsaobis is characterized by rugged, hilly terrain with sparse vegetation, and seasonal riverbeds with patches of denser riparian trees and shrubs, within an arid, desert environment. We followed two troops of baboons (J troop, *n* = 65; and L troop, *n* = 86) that have been habituated to the presence of observers on foot and are individually identifiable, allowing excellent thermal images to be taken *in situ*. Data for this project were collected between May and July 2023. Long-term data regarding individuals’ sex and age were available from the Tsaobis Baboon Project database with age–sex breakdown shown in table 1. Dominance ranks were calculated from ad libitum observed dominance events such as chasing, supplanting, attacking, displacing and threatening behaviours using the inconsistency and strength of inconsistency method in the EloRating package [27], which identifies the most linear hierarchy by minimizing the number and strength of dominance inconsistencies. Relative rank represents the proportionate position of an individual within the troop’s dominance hierarchy, scaled between 0 and 1, where higher values indicate higher dominance relative to the group and lower values reflect lower dominance. All rank measures used throughout the study refer to this relative rank scale. Grooming interactions were collected ad libitum as observers moved through the troops. To ensure independence, grooming between two individuals was recorded only once in 30 min, even if grooming direction changed between the dyad. Social affiliation was then calculated as the proportion of grooming given by individual A to individual B (A–B relationship) to the total grooming interactions A gave to all troop members [28]. This is a directional measurement, which means that the value for A–B is not the same as the value for B–A (unless the relationship is equal).

**Table 1.** Breakdown of the age–sex classes in J and L troops.

	adult male	subadult male	juvenile male	infant male	adult female	juvenile female	infant female
J troop	3	3	14	7	23	5	10
L troop	12	6	9	7	27	12	13

### 2.2. Image acquisition and temperature measurements

Thermal images were collected from individuals within J and L troops by E.J. using an RS PRO thermal imaging camera with RS calibration (384 px × 288 px) (RS Components Ltd., London). The thermal imager’s spectral range is from 8 to 14 μm, and its thermal sensitivity is less than 50 mK at 30°C with manual focus. The lens has a field of view of 41.5° × 31.1°/0.5 m with a resolution of 1.89 mrad. Emissivity was set at 0.95 on the camera, aligning with the standard emissivity of bare skin [29]. The supplied software automatically accounted for reflected ambient temperature. The camera auto-calibrated before an image was taken, while the observer manually focused on the subject’s nasal area for the best image quality. We aimed to take images of the baboons’ facial areas when they directly faced the camera, wherever possible. A facial angle of more than 45° between the direction the baboon was facing and the position of the camera was noted within the dataset. Images were only taken when the baboons were in the shade to avoid the effect of direct sunlight and, where possible, when the observer was also in the shade. Owing to the sparsely vegetated environment of the field site, fully shaded areas were limited, and finding optimal conditions for thermal photography occasionally required extended waiting periods or adapting to subtle shifts in the troop’s position. When possible, we prioritized individuals who were fully in the shade, but we acknowledge that in some cases, baboons may have recently moved from sun-exposed to shaded areas before the image was taken. This could potentially influence surface temperature, particularly in facial regions. We attempted to minimize this risk by allowing individuals to remain in an activity (e.g. grooming, foraging and resting) for at least 10 min before taking an image, thereby allowing time for transient thermal effects—such as those caused by prior sun exposure—to stabilize [30]. Images were also attempted to be taken when there was no strong wind owing to the wind’s cooling effect from convection heat loss [31]. A separate thermometer (Omega model OM-HL-SP-TH) measured ambient temperature within each observation for environmental variable control. Owing to the desert environment, relative humidity was always low, and these data were not recorded. We recorded other environmental conditions, including any sunlight on the subject or observer and strong wind for individual images. Images were discarded if there was strong wind or intense direct sunlight to ensure that the ambient condition did not affect data accuracy.

We captured images of baboons during activities in line with our aims, i.e. while giving and receiving grooming, foraging and resting. Grooming was defined as manipulating the fur of another individual; the groomer refers to the individual giving grooming, while the groomee is the individual receiving grooming. Giving grooming was recorded when the focal individual was grooming, receiving grooming was recorded when the focal individual’s fur was being manipulated by another individual. Foraging was defined as searching for, picking up and ingesting food items. In arid environments such as our field site, where food is patchily distributed and monopolizable, lower ranking individuals are often displaced from feeding sites or must forage in lower quality areas, resulting in greater competition and reduced foraging efficiency [32,33]. Resting was defined as sitting or lying while not moving (but not asleep, i.e. the individual had their eyes open). The observer aimed to maintain a distance of approximately 5 m from the focal subject and capture a minimum of three repeated thermal images during each session to assess repeatability. Grooming partners were recorded for grooming sessions. The audience was recorded as all individuals within 5 m of the focal individual.

**Figure 1.**
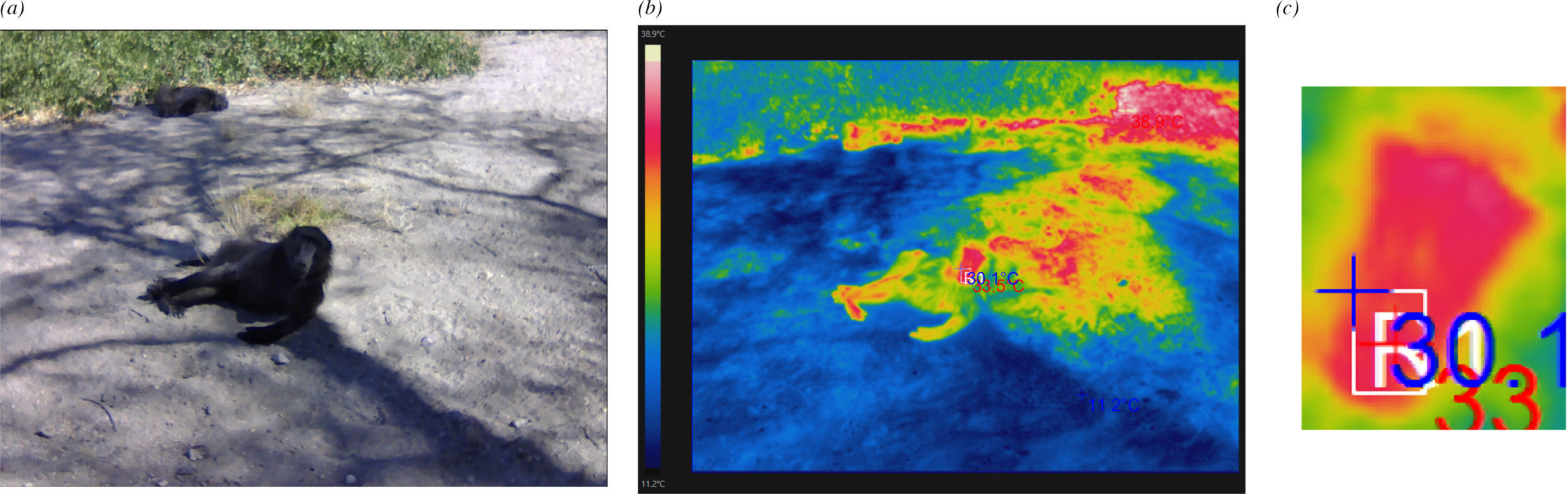
(*a*) An example of the original photo image from an adult male baboon resting. (*b*) A thermal image with temperature scale of the same image. (*c*) Zoomed-in thermal image of the selected area of interest. In the thermal image, the white outline around the nasal region depicts the area of interest, including nostrils, nose ridge and adjacent areas. The blue text next to the area of interest indicates the lowest temperature and the red the highest temperature in the selected region.

The images were processed with the ThermoView Pro software. As in previous studies [34], the region of interest (ROI) was the nasal area of the focal individual and was manually delineated by E.J. using a rectangular selection tool within the software (figure 1). This area was positioned to capture the nasal bridge while avoiding surrounding fur or shaded areas. Although the size of the ROI varied slightly across images, it was applied only when the nasal area was clearly distinguishable and unobstructed. To ensure consistency in placement, facial landmarks such as the nostrils and eye position were used to guide ROI alignment across images. Previous studies have shown that factors such as airflow from nostrils and minor head movements do not significantly affect facial temperature readings in thermal imaging [3–5], supporting the reliability of our approach under naturalistic conditions. To ensure consistency, all ROIs were manually delineated by a single trained observer (E.J.). While we did not conduct an inter-rater reliability test as it was not relevant in our design, we acknowledge that doing so could further strengthen reproducibility and recommend it as a valuable step for future studies.

### 2.3. Data analyses and statistical methods

We performed the following analyses in line with our four research aims. First, to determine whether thermal image data collected under desert field conditions were repeatable, we calculated the repeatability of our thermal measurements of the same baboon within a short time interval. Repeatability is a measure of the consistency of repeated measurements on the same subject. Following Tkaczynski *et al*. [24], we estimated repeatability with a Gaussian data type using the ‘rptR’ package in R [35]. The analysis was run with 10 000 bootstrap replicates (*n*_boot_ = 10 000) and 10 000 permutations (*n*_permut_ = 10 000).

Second, to test whether nasal temperature differed across different activities, and following the approach of de Vevey *et al*. [2], we ran a linear mixed model (LMM) with nasal temperature as the response variable and activity (groom, forage and rest) as the main predictor using the *lmer* function in the ‘lme4’ package [36]. The number of individuals within a 5 m radius of the focal subject (hereafter referred to as audience number) was also included here as a predictor of interest to address aim 4a (see below). We controlled for ambient temperature, individual relative rank and age group as fixed effects. Session ID and individual ID were further fitted as random effects to control for repeated measures within a photographic ‘session’ and of individuals across the study. Because large males outrank females, individual relative rank is confounded with sex, and we did not include sex as an independent control variable (*ρ* = 0.418, *p* < 0.001). Infants were excluded from all statistical analyses owing to the small number of observations recorded in this age group (*n* = 26 thermal images).

Third, to test whether nasal temperature differed depending on whether a baboon was giving or receiving grooming, we ran an LMM on the grooming subset of the data (i.e. excluding all photographs of baboons engaged in other activities than grooming), with nasal temperature as the response variable and activities with grooming direction (groomee or groomer) as the main predictor. As for the previous LMM, we controlled for ambient temperature, individual relative rank, age group, session ID and individual ID.

Finally, to test whether there was a correlation between the composition of the audience and focal subject’s nasal temperature, we used three different measures of the audience to address our three predictions: (4a) the total number of individuals within 5 m (total number of audience); (4b) the relative rank of the highest ranking non-focal individual in the audience; and (4c) the median of the social affiliation indices from the focal subject to each member of the audience. Aim 4a was addressed using the model developed for aim 2 (see above). We ran two further LMMs to address aims 4b and 4c. For sessions in which no other individuals were within 5 m, audience size was coded as zero. In these cases, the audience rank and affiliation variables were coded as NA, and the corresponding data points were excluded from models where those variables were the predictor of interest, to ensure valid comparisons within each model framework. For the aim 4b model, which used audience rank as a predictor, infants were additionally excluded when they could not be reliably identified or did not have rank data. For the aim 4c model, which used social affiliation as a predictor, audience bond scores were set to NA when no grooming data existed between the focal individual and audience members; these cases were excluded from the model to ensure valid comparisons.

In model 4b, we selected the rank of the highest ranking audience member because it best captures the dominance influence of the most socially influential individual present, aligning with the use of alpha male presence as a key variable in de Vevey *et al.* [2]. We also tested an alternative fixed effect variable: the relative rank difference between the focal subject and the highest ranking audience member, but this variable was highly correlated with the highest audience rank and did not improve model fit or interpretability. In model 4c, although the audience composition is heterogeneous, the median measure was chosen to represent the central tendency of social affiliation, mitigating the influence of outliers and providing a robust estimate of overall social context, cf. the rank of the highest ranking individual.

Because it was statistically more likely that higher ranking individuals would be found in larger groups, we also tested whether there was a correlation between the size of the audience (when at least one individual was present within 5 m) and the relative rank of the highest ranking audience member using a Spearman rank correlation. These variables were moderately correlated (*ρ* = 0.365, *p* < 0.001). While the audience size and audience rank variables were not included in the same model, they were both significant predictors of nasal temperature (see Results). To understand whether the effects of audience size and rank were distinct or reflected overlapping social context, we explored their correlation to determine whether high-ranking individuals were systematically more likely to be present in larger audiences. To do so, we ran two separate models, one using audience size and the other using highest audience rank, on the same subset of data (excluding cases where no audience was present, as audience rank could not be defined in those instances), and compared the models’ Akaike information criteria (AIC) values. As in the previous models, we included ambient temperature, individual relative rank, age group, session ID and individual ID as controls.

Model diagnostics were performed on all models to assess key statistical assumptions of normality of residuals, homoscedasticity and multicollinearity. Model performance was inspected using the *performance* package [37]. No violations of model assumptions were detected. Variance inflation factors (VIF) were computed using the *vif* function to check for multicollinearity among predictors. No predictor had a VIF above 5, indicating little collinearity [38]. Model fit was evaluated further using marginal and conditional *R*^²^ values, which indicated satisfactory model performance. Full-null model comparisons were conducted with analysis of variance (ANOVA) tests to evaluate the overall significance of the variable of interest [39]. Posthoc pairwise comparisons were conducted using the *emmeans* package [40], which conducted adjusted pairwise tests controlling for Type I errors.

## 3. Results

We collected *n* = 1626 thermal images of individuals from 388 sessions in J and L troops (*N*_J_ = 613, *N*_L_ = 1013). A total of 105 individuals were photographed (*N*_J_ = 46, *N*_L_ = 60); we note that the total number of individuals (105) is one fewer than J + L (46 + 60) as one subadult male moved between troops during the study period and was photographed in both troops. A mean of 4.21 ± 2.06 (s.d., range = 1–13) images were taken per session.

### 3.1. Aim 1: repeatability

Mean nasal temperatures were highly repeatable within each session of imaging (*R* = 0.961 (s.e. = 0.003, 95% confidence interval (CI) = [0.953, 0.967], *p* < 0.001; electronic supplementary material, figure S1).

### 3.2. Aim 2: effects of activity

**Figure 2.**
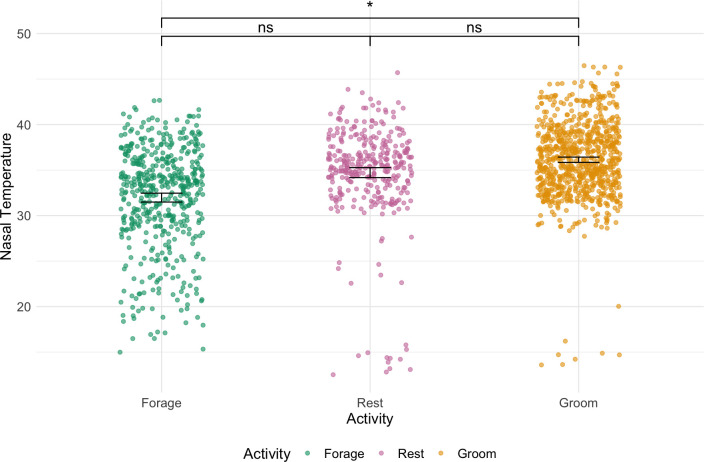
Nasal temperature by activity with model predictions and error bars. The figure displays nasal temperatures for different activities: forage (green), rest (pink) and groom (orange). Jittered raw data points are colour-coded by activity, with error bars indicating bootstrapped confidence intervals. Significant differences calculated from *emmeans* are marked with asterisks at the top.

In our analysis of nasal temperature across different activities, we found that the full model including activity explained significantly more variation than the null model ($\chi_{3}^{2}$ = 16.07, *p* = 0.001). Specifically, nasal temperature was significantly higher while grooming compared with foraging (forage – groom: estimate = −1.11, s.e. = 0.45, *p* = 0.015), whereas nasal temperature did not differ between resting and foraging (forage – rest: estimate = −0.64, s.e. = 0.53, *p* = 0.227). The difference between resting and grooming was not significant (rest – groom: estimate = −0.47, s.e. = 0.48, *p* = 0.327; figure 2). Additionally, juveniles showed a statistically significant lower nasal temperature compared with adults (estimate = −1.44, s.e. = 0.49, *p* = 0.004; table 2).

**Table 2.** Aim 2 model results for activities predicting nasal temperature (*N* obs: 1583; sessions: 375; individuals: 98). (Note: statistically significant effects (*p* < 0.05) are shown in bold.)

predictor	estimate	s.e.	**95% CI**	*t*-score	***p***‐**value**
intercept	11.01	1.42	[8.23, 13.80]	7.77	<0.001
env temp	**0.79**	**0.05**	**[0.70, 0.88]**	**17.15**	**<0**.**001**
activity (rest)^a^	0.64	0.53	[−0.40, 1.68]	1.21	0.227
activity (groom)^a^	**1.11**	**0.45**	**[0.22, 2.00]**	**2.44**	**0.015**
audience number	**−0.40**	**0.14**	**[−0.67, −0.13]**	**−2.90**	**0.004**
relative rank	−0.09	0.63	[−1.33, 1.15]	−0.14	0.885
age group (juvenile)^b^	**−1.44**	**0.49**	**[−2.41, −0.47]**	**−2.91**	**0.004**
age group (subadult)^b^	−0.56	0.59	[−1.72, 0.61]	−0.94	0.349

^a^ Reference category = forage.

^b^ Reference category = adult.

### 3.3. Aim 3: effects of grooming roles

We tested whether there was a difference in subjects’ nasal temperatures between being a groomer or groomee. There was no difference between the nasal temperature of the groomer and groomee (estimate = 0.43, s.e. = 0.50, *p* = 0.390; electronic supplementary material, table S1). There were also no effects of age group or relative rank on nasal temperature in this model.

### 3.4. Aim 4: audience effects

**Figure 3.**
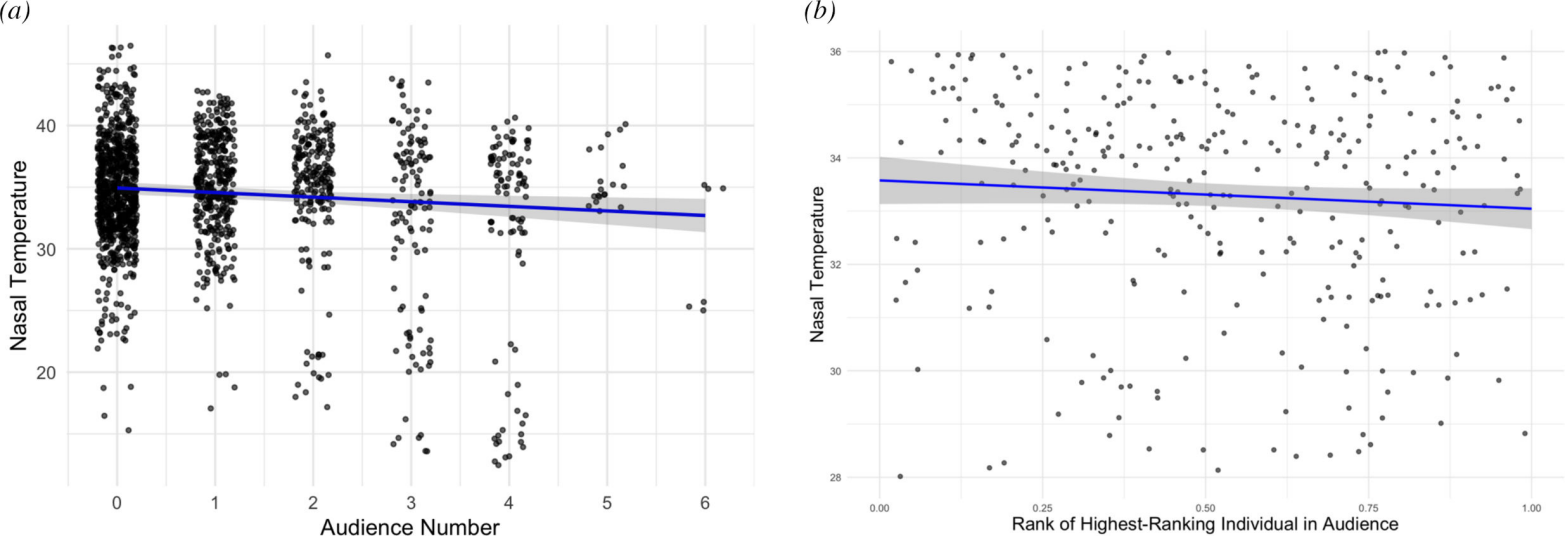
Social effects on nasal temperatures. (*a*) Relationship between audience number and subject nasal temperature. (*b*) Relationship between rank of the highest ranking audience member and subject nasal temperature. In both plots, points represent raw data, and the line indicates a linear regression fit with a 95% confidence interval.

We tested for the relationship between a subject’s nasal temperature and three possible measures of a subject’s audience. Both the audience number (table 2; figure 3*a*) and the rank of the highest ranking audience member (table 3; figure 3*b*) had a significant negative correlation with the subject’s nasal temperature while controlling for other factors. To determine whether one variable explained more variation and thus was a ‘better’ predictor of nasal temperature, we compared the AIC of these models using the same dataset. We found no difference in the models’ predictive quality (audience number model: AIC = 2889.23; highest audience rank model: AIC = 2889.80). Finally, testing the relationship between social bond and subject’s nasal temperature, we found no statistically significant relationship (estimate = 1.61, s.e. = 2.09, *p* = 0.441) between subjects’ nasal temperatures and the median social affiliation with audience members (electronic supplementary material, table S2).

**Table 3.** Model results for highest audience rank and nasal temperature (*N* obs: 762; sessions: 197; individuals: 79). (Note: Statistically significant effects (*p* < 0.05) are shown in bold.)

predictor	estimate	s.e.	95% CI	*t*-score	*p*-value
intercept	6.20	1.86	[2.54, 9.86]	3.33	<0.001
env temp	**0.99**	**0.06**	**[0.87, 1.10]**	**17.18**	**<0**.**001**
highest audience rank	**−1.87**	**0.81**	**[−3.47, −0.27]**	**−2.30**	**0.022**
activity (rest)^a^	−0.96	0.64	[−2.21, 0.29]	−1.51	0.131
activity (groom)^a^	0.17	0.58	[−0.97, 1.30]	0.29	0.774
relative rank	0.17	0.99	[−1.77, 2.10]	0.17	0.866
age group (juvenile)^b^	**−2.40**	**0.76**	**[−3.89, −0.91]**	**−3.17**	**0.002**
age group (subadult)^b^	−0.15	0.88	[−1.88, 1.59]	−0.17	0.866

^a^ Reference category = forage.

^b^ Reference category = adult.

## 4. Discussion

The primary objective of the study was to examine how nasal temperature in wild baboons was influenced by context, including individual activities and audience effects, and whether such results align with presumed emotional valence associated with the activities. We found that nasal temperature measurements within observations were highly repeatable, suggesting that the measures had high precision, as has been found in previous studies [34,41] and in line with our prediction 1. We also found that baboons who were foraging had lower nasal temperatures than those who were grooming, providing support for predictions 2a and 2b, albeit neither foraging nor grooming temperatures differed from resting temperatures. Also, in line with our predictions 4a and 4b, subjects with a larger audience and with higher ranking individuals in the audience showed lower nasal temperatures. However, contrary to our predictions 3 and 4c, there were no effects of grooming role or median social affiliation of the audience to the subject on their nasal temperature. Below we discuss the implications of these findings for the use of thermal imaging in behavioural studies of wildlife, and their potential application to the study of welfare or emotions in animals.

In line with our activity predictions (predictions 2a, 2b), baboons had higher nasal temperatures when grooming compared with foraging. This may suggest a more relaxed emotional state during grooming indicated by subcutaneous vasodilation [10]. While elevated nasal temperature could, in principle, reflect different forms of positive arousal, including sexual interest [4,34], we interpret the increase in this context as indicative of relaxation or social calm. This is because grooming in chacma baboons is an affiliative interaction in most cases [33,42]. Our findings are broadly consistent with de Vevey *et al.* [2], who also found that grooming (a socio-positive interaction) was associated with higher nasal temperatures than foraging (a potentially competitive activity), but that neither activity differed significantly from resting (baseline). This similarity strengthens the interpretation that grooming and foraging elicit distinct physiological responses, even if resting remains a more variable baseline condition. While we interpret these thermal patterns as reflecting underlying physiological arousal and emotional valence, we do so cautiously. As in previous work, we acknowledge that thermal responses are indirect proxies of emotion and must be interpreted within their behavioural and social context.

In contrast to grooming, we observed lower nasal temperatures during foraging. Chacma baboons have a strongly linear and strictly enforced dominance hierarchy, which could make foraging socially stressful [1,3,11], resulting in heightened arousal and negative emotional valence, potentially linked to sympathetic activation [43]. However, this interpretation is complicated by the finding that neither grooming nor foraging nasal temperatures differed from resting nasal temperatures. One possible explanation is that the resting category, as defined in our model, encompasses a wide range of internal states and social contexts. While we accounted for specific social factors such as audience number and the presence of higher raking individuals in our model, the residual variability within resting contexts, such as differences in proximity to social partners or variations in alertness could still have an effect. This variability could have contributed to high within-activity variation and may have limited our ability to detect statistically significant differences. While our models included audience size and relative rank as fixed effects, we did not explore how their influence might vary across different behavioural contexts to avoid over-parameterization. Future work could assess whether audience effects are modulated by activity type—for example, whether the presence of higher ranking individuals is more stressful during foraging than during grooming.

Our analysis of activities also indicated an age effect: juveniles exhibit lower nasal temperatures than adults. The lower nasal temperatures of juveniles may be influenced by physiological differences, such as a smaller surface-area-to-volume ratio, making heat retention more challenging [44]. Furthermore, juveniles may exhibit less vasodilation owing to higher activity levels or more frequent activation of the sympathetic nervous system, possibly because of their increased vulnerability and smaller size. Consistent with this pattern, younger primates tend to show higher baseline cortisol levels and a more rapid stress response compared with older individuals [45]. Behavioural differences, such as juveniles grooming less frequently than adults [46], could also contribute to this effect. This supports the idea that juveniles are still undergoing physiological development and remain more sensitive to environmental and social stimuli. Indeed, developmental studies in primates have demonstrated that the neuroendocrine systems, including the hypothalamic–pituitary–adrenal axis, are still maturing in younger individuals, leading to heightened physiological reactivity and less efficient regulation of stress responses compared with adults [47,48].

Contrary to our prediction about grooming roles (prediction 3), there were no significant differences observed in nasal temperatures between groomers and groomees. This may emphasize the complexity and variability of grooming interactions across different contexts. On the one hand, previous studies have shown that groomees were generally more relaxed, as indicated by reduced heart rate, which aligns with the stress-reduction hypothesis of grooming reception [13,14]. On the other hand, one study has reported that groomers were more relaxed than groomees, suggesting that the act of giving grooming may also confer psychological benefits under certain conditions [49]. Our findings also diverge from those of de Vevey *et al.* [2], who used thermal imaging to show that chimpanzees had significantly lower nasal temperatures when giving grooming compared with receiving or mutually grooming. They also found that chimpanzee nasal temperatures were significantly lower when grooming was given, unreciprocated, or to the alpha male. These findings suggest that the social identity of the grooming partner, including their dominance status and whether grooming was reciprocated, can shape the physiological response to grooming. While we did not include such detailed partner-specific variables in our analysis, future research may benefit from incorporating interaction terms that account for partner rank, social bond strength and asymmetries in grooming exchanges. This would allow for a more fine-grained understanding of how grooming direction interacts with social context to influence physiological outcomes.

In line with two of our predictions about audience effects (predictions 4a, 4b), baboons with larger audience or higher ranking individuals in the audience had lower nasal temperatures. One interpretation of this finding is the presence of a larger audience, and/or higher ranking individuals represent more competitive contexts, resulting in the lower nasal temperatures typically associated with competition and/or stress [34]. The lack of difference in predictive quality between the audience number model and the highest audience rank model, however, implies that neither variable conclusively drives this phenomenon, and that both variables could be equally important in affecting nasal temperature. While we modelled the highest audience rank using the rank of the individual with the highest Elo-score present, we also considered alternative formulations, including the difference between the focal individual’s relative rank and that of the highest ranking audience member. This approach was highly correlated with the highest audience rank model and did not improve model fit or interpretability in our case, but relative rank differences may be relevant in other contexts or species as a direction for future research.

Although relatively few studies have applied thermal imaging to investigate audience effects in wild primates, recent work in wild chimpanzees offers meaningful parallels. Nasal temperatures decreased during competitive events and increased during affiliative interactions, with audience composition significantly moderating these effects [2]. For example, the presence of the alpha male reduced the positive thermal signature typically associated with affiliation, while the presence of females buffered the negative effects of competition. Similarly, chimpanzees exhibited lower nasal temperatures when feeding on contested resources (meat versus figs), and that nasal temperature responses were shaped by audience structure, particularly the number, dominance status and social bond strength of nearby males [1]. These findings reinforce the idea that thermal responses in primates are sensitive not only to the nature of the activity but also to the specific social environment in which it occurs. Our own findings in chacma baboons support this pattern, suggesting that both audience size and the presence of high-ranking individuals may shape the physiological—and potentially emotional—experience of social behaviour. Together, these studies highlight the use of thermal imaging for investigating how primates monitor and respond to dynamic social contexts in real time.

We found no significant relationship between the subject’s nasal temperature and median social affiliation with the audience (prediction 4c). Previous research using thermal imaging in wild chimpanzees has demonstrated that social bonds can meaningfully modulate thermal responses. For example, the strength of social bonds with nearby males influenced thermal responses of chimpanzees during feeding, particularly under competitive conditions [1]. Considering this, one possibility for the lack of an effect in our study could be how social bonds were quantified and may suggest that the median social bond may not be the most appropriate measure. Accurately capturing the role of social relationships in primate physiology is inherently complex, as different network metrics capture different dimensions of affiliative ties and social dynamics [50,51]. An alternative measure could capture variation in social context, for instance, by reflecting where a focal individual was surrounded by both highly bonded and less bonded group members. Future work may benefit from employing more sensitive or granular metrics, including maximum bond strength, proximity-weighted affiliation or full audience composition, to better evaluate how social bonds influence physiological responses and emotional states in complex social environments. Alternatively, our result could be real, and, contrary to chimpanzees, reflect a true absence of influence of social bonds on thermal responses in baboons.

One major limitation of the current study is the inferential nature of the links between nasal temperature and emotions. Although some literature supports the connection [4,5], this relationship remains a topic of ongoing research and debate. This limitation emphasizes the need for cautious interpretation of our findings and highlights the importance of further research to establish more definitive connections. Wherever possible, adopting a multimodal approach to the study of transient behavioural states by combining thermal imaging with other biometric data—such as heart rate monitoring, hormone assays and gene expression studies—or more detailed and targeted behavioural data can provide a more comprehensive understanding of wildlife psychology and welfare [52]—albeit very costly and labour-intensive. By contrast, thermal imaging offers a non-invasive, cheaper and less labour-intensive alternative to methods that require the collection of potentially difficult-to-obtain blood, urine or faecal samples. Additionally, unlike traditional hormone measures that may not capture rapid physiological changes, thermal imaging provides real-time data on immediate responses. Thermal imaging, being cost-efficient and non-invasive, can fill this real-time niche effectively. However, thermal imaging may be less appropriate for evaluating long-term welfare or chronic stress. Therefore, integrating multiple methods allows us to capture both immediate and long-term physiological and psychological responses, offering a more holistic approach to understanding animal welfare.

Thermal imaging has extended our understanding of physiological changes associated with emotions in humans [53] and is now being applied in ethological studies of animal behaviour and welfare [1,2,17,18]. Our findings contribute to this emerging field by demonstrating that, even in the wild, where it is impossible to control the social and physical environment, thermal patterns can reflect social and behavioural context in meaningful ways. Specifically, we observed elevated nasal temperatures during grooming, an affiliative behaviour typically associated with positive social engagement, and reduced temperatures during foraging, in the presence of a large audience or when higher ranking individuals were nearby. These patterns are consistent with shifts in autonomic nervous system activity that may underlie emotional states, suggesting that thermal imaging can offer a valuable, non-invasive tool for investigating emotional processes in non-verbal animals.

More broadly, our results speak to the long-standing challenge of assessing animal emotions through observable indicators. Behaviour alone is often an ambiguous proxy for emotion, especially in species where overt displays may be shaped by evolutionary pressures or social masking. Thermal imaging offers a novel bridge between external behaviours and internal physiological states, allowing researchers to infer patterns of arousal and—more cautiously—emotional valence in ecologically valid contexts.

## Data Availability

Data are available at [54]. Supplementary material is available online [55].
